# A rare natural lipid induces neuroglobin expression to prevent amyloid oligomers toxicity and retinal neurodegeneration

**DOI:** 10.1111/acel.13645

**Published:** 2022-06-03

**Authors:** Henry Patrick Oamen, Nathaly Romero Romero, Philip Knuckles, Juha Saarikangas, Marta Radman‐Livaja, Yuhong Dong, Fabrice Caudron

**Affiliations:** ^1^ School of Biological and Behavioural Sciences Queen Mary University of London London UK; ^2^ Friedrich Miescher Institute for Biomedical Research Basel Switzerland; ^3^ Helsinki Institute of Life Science, HiLIFE, University of Helsinki Helsinki Finland; ^4^ Research Programme in Molecular and Integrative Biosciences, Faculty of Biological and Environmental Sciences University of Helsinki Helsinki Finland; ^5^ Neuroscience Center, University of Helsinki Helsinki Finland; ^6^ IGMM, Univ Montpellier, CNRS Montpellier France; ^7^ SunRegen Healthcare AG Reinach Switzerland

**Keywords:** amyloid, neurodegeneration, neuroglobin, neuroprotection, protein aggregation, tripentadecanoin, yeast aging, Yhb1

## Abstract

Most neurodegenerative diseases such as Alzheimer's disease are proteinopathies linked to the toxicity of amyloid oligomers. Treatments to delay or cure these diseases are lacking. Using budding yeast, we report that the natural lipid tripentadecanoin induces expression of the nitric oxide oxidoreductase Yhb1 to prevent the formation of protein aggregates during aging and extends replicative lifespan. In mammals, tripentadecanoin induces expression of the Yhb1 orthologue, neuroglobin, to protect neurons against amyloid toxicity. Tripentadecanoin also rescues photoreceptors in a mouse model of retinal degeneration and retinal ganglion cells in a Rhesus monkey model of optic atrophy. Together, we propose that tripentadecanoin affects p‐bodies to induce neuroglobin expression and offers a potential treatment for proteinopathies and retinal neurodegeneration.

## Graphical abstract

The natural lipid tripentadecanoin induces the expression of Yhb1 in budding yeast and its orthologue Neuroglobin in mammals. Yhb1 induction prevents the formation of age‐induced protein aggregates and delays aging in yeast. Neuroglobin induction prevents the toxicity of diverse amyloid oligomers in neurons and chemically or genetically induced retinal degeneration in animals.

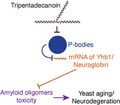

## 1 INTRODUCTION

Age‐associated neurodegenerative diseases, including Alzheimer's, Parkinson's and prion diseases are linked to the toxicity caused by protein misfolding, particularly into amyloid fold (Wells et al., 2021). For example, brains of Alzheimer's disease patients commonly display β‐amyloid and/or neurofibrillary tangles of tau that accumulate outside and inside neurons, spread between cells and thereby disrupt normal cell functions. These diseases are becoming increasingly prevalent and current curative therapies are insufficient. Therefore, there is an urgent need to develop therapeutic agents that counteract amyloid toxicity.

Protein homeostasis seems to be equally important in non‐dementia degenerative diseases, affecting for example the retina during aging (Athanasiou et al., 2013; Leger et al., 2011; Tzekov et al., 2011). These data suggest that understanding protein homeostasis and finding mechanisms to improve it during aging may offer a therapeutic window for multiple diseases.

The budding yeast *Saccharomyces cerevisiae* has been a useful model to understand how cells can counteract protein aggregation toxicity. Heterologous expression of aggregation‐prone proteins involved in human diseases or analysis of their yeast orthologues has revealed mechanistic insights into Friedreich's Ataxia (Babcock et al., 1997), Amyotrophic lateral sclerosis (Couthouis et al., 2011; Jovičić et al., 2015), Huntington's disease (Ripaud et al., 2014), Alzheimer's disease (Treusch et al., 2011) and enabled identification of promising compounds for treating Parkinson's disease (Tardiff et al., 2013).

In addition, yeast provides a useful model for cellular senescence during replicative aging, which is the increase in mortality with the number of daughter cells a yeast mother cell has produced. In old cells, age‐induced protein aggregates form in the mother cell (Saarikangas & Barral, 2015). The presence of these protein aggregates limits yeast replicative lifespan and is therefore a good reporter for assessing aging phenotypically. Age‐induced protein aggregates can be visualized using the protein disaggregase Hsp104 fused to a green fluorescent protein (GFP) or the Hsp70 family member Ssa1 fused to GFP as reporters. They form a single focus in the cytoplasm (Saarikangas et al., 2017; Saarikangas & Barral, 2015) and represent deposits that accumulate damaged proteins (Aguilaniu et al., 2003; Erjavec et al., 2007; Saarikangas & Barral, 2015). However, which specific damaged proteins accumulate in these deposits is unknown.

Here, we present the identification of the glycerolipid tripentadecanoin as a potent molecule counteracting protein aggregation toxicity in different models.

## 2 RESULTS

**FIGURE 1:**
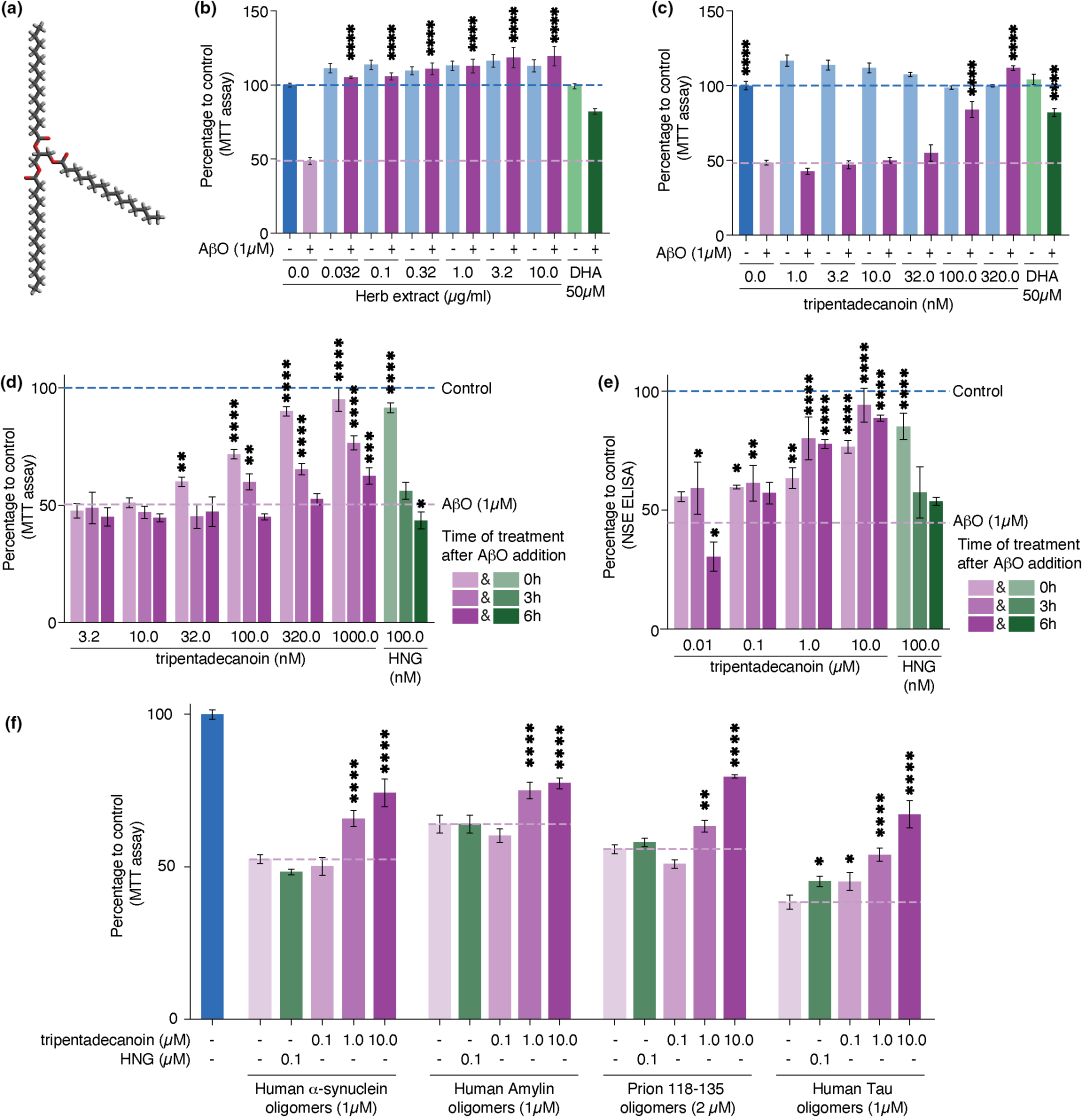
Ophioglossum whole extract and tripentadecanoin protect neurons against amyloid toxicity. (a) Structure of tripentadecanoin. (b–c) quantification of mouse primary cortex neurons viability (MTT assay) after exposure to AβO (1 μM) and pre‐treatment with Ophioglossum extract or docosahexaenoic acid (DHA) (b) or pre‐treatment with tripentadecanoin or DHA (c). (d) Quantification of mouse primary cortex neurons viability (MTT assay) after treatment with tripentadecanoin or Humanin (HNG) 0, 3 or 6 h after exposure to AβO (1 μM). (e) Quantification of human‐induced pluripotent stem cells derived neurons viability (NSE assay) after treatment with tripentadecanoin or HNG 0, 3 or 6 h after exposure to AβO (1 μM). (f) Quantification of mouse primary cortex neurons viability (MTT assay) after treatment with tripentadecanoin or HNG 3 h after exposure to human α‐synuclein oligomers (1 μM), human amylin oligomers (1 μM), prion Protein_118‐135_ oligomers (2 μM) or human tau oligomers (1 μM). *p*‐values were obtained from ANOVA comparing to the toxin only treatment (A‐E); * < 0.05; ** < 0.01; *** < 0.001; **** < 0.0001

*Ophioglossum thermale* is a medicinal herb used in traditional Chinese medicine as a rescuing treatment for snake bite toxicity and occasionally for conditions of neuronal atrophy. The glycerolipid tripentadecanoin (Figure 1a) was reported as one of *Ophioglossum*'*s* extract components (Guo‐Wei et al., 2011). We tested the effect of *Ophioglossum* whole extracts and tripentadecanoin on neurons challenged by β‐amyloid_1‐42_ oligomers (AβO) toxicity. Mouse primary cortex neurons were incubated 48 h with different concentrations of the herb whole extract, tripentadecanoin or docosahexaenoic acid (DHA, 22:6 n‐3) as a positive control (Florent et al., 2006) before addition of 1 μM AβO. After 24 h, cell viability was assayed by MTT‐colorimetry and demonstrated that the herb extract provided neuroprotection against AβO at all concentrations tested (Figure 1b). Tripentadecanoin provided neuroprotection from 100 nM and higher concentrations (Figure 1c), indicating that it is most probably the main active compound that conferred the neuroprotective effect of *Ophioglossum* extract. We note however that other compounds than tripentadecanoin in the herb extract may also contribute to the herb extract effect. Testing other lipids with similar structures using this assay demonstrated that the neuroprotection effect was specific to tripentadecanoin, although linoleic acid provided some neuroprotection (Figure S1). We then asked whether tripentadecanoin was also neuroprotective when added concurrently or after AβO. For these experiments, we used Humanin (HNG) as a positive control (Hashimoto et al., 2001), since DHA does not protect neurons in these conditions. Tripentadecanoin was added at 0, 3 or 6 h after AβO. Remarkably, tripentadecanoin was highly neurorescuing (320 nM and 1 μM) after 3 h and neurorescuing after 6 h (Figure 1d). Next, we tested tripentadecanoin neuroprotective effects on human cells. We used neurons derived from induced pluripotent stem cells and challenged them with AβO (1 μM). Tripentadecanoin or HNG was added after 0, 3 or 6 h and cell viability was assayed by a neuron‐specific enolase assay, as an alternative assay to support our results obtained with the MTT assay. Compared to mouse neurons, tripentadecanoin protected human neurons with a higher efficiency (Figure 1e). We conclude that *Ophioglossum* extract and tripentadecanoin are neuroprotective and neurorescuing pharmaceutical agents against AβO‐induced toxicity. To test if the protective effect was specific to AβO or more broadly applicable to toxic amyloid oligomers, we tested its effects in mouse primary cortex neurons challenged with human α‐synuclein oligomers (1 μM), human amylin oligomers (1 μM), Prion Protein_118‐135_ oligomers (2 μM) and human Tau oligomers (1 μM). Importantly, tripentadecanoin displayed neuroprotective effects for all these toxic proteins (Figure 1f) suggesting a common downstream target.

**FIGURE 2:**
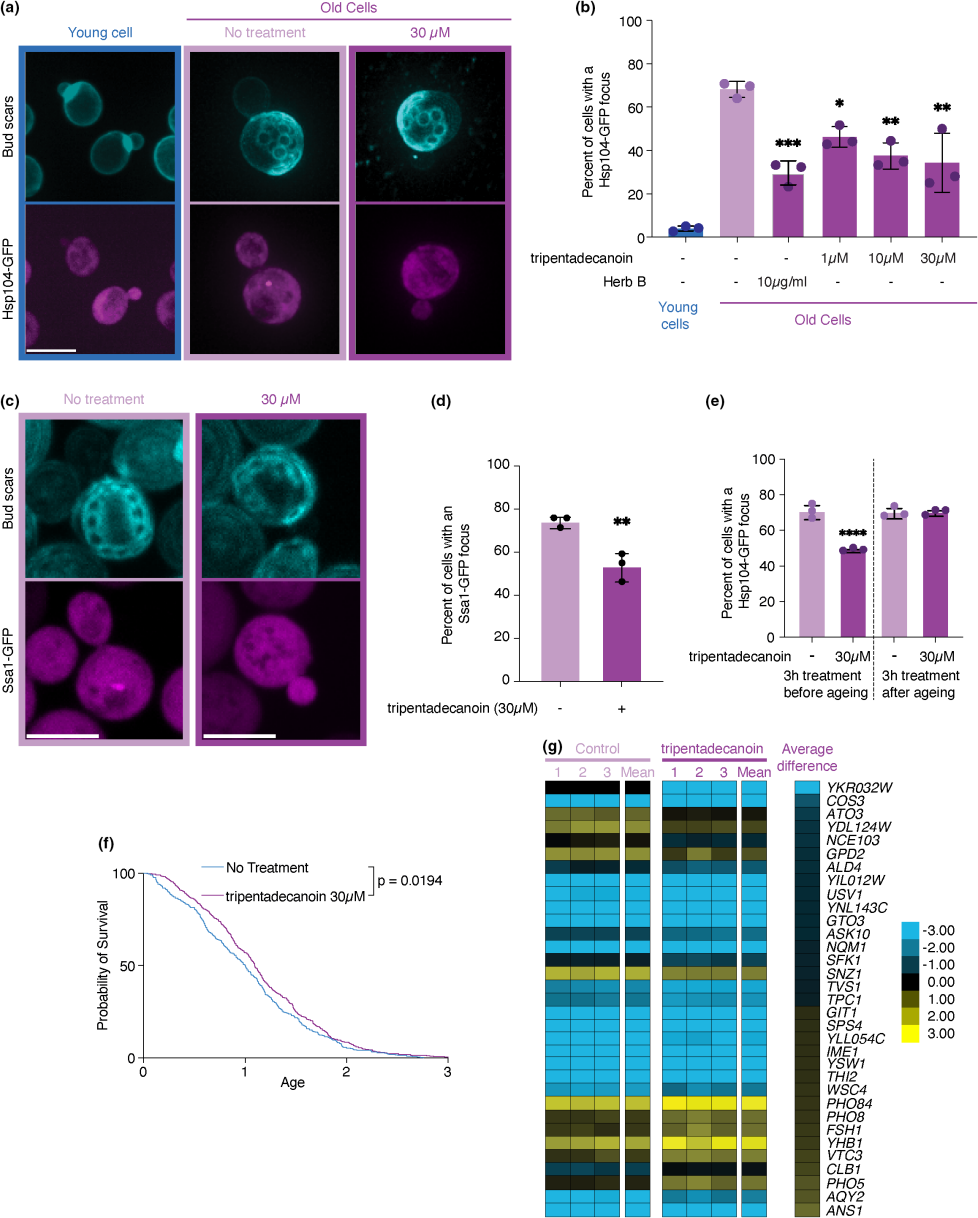
Tripentadecanoin prevents the formation of age‐induced protein deposits in budding yeast. (a) Representative images of a young cell, or old cells ± tripentadecanoin (30 μM). Upper panels show cells stained with fluorescent brightener 28 to reveal bud scars. Lower panels display the Hsp104‐GFP signal. Scale bar = 5 μm. (b) Percentage of cells with an Hsp104‐GFP focus. Mean ± SD. Dots represent independent experiments (N ≥ 75 cells). *p* values are adjusted *p* values from an ANOVA comparing to untreated old cells *<0.05; **<0.01; ****<0.0001. (c) Representative images of old cells ± tripentadecanoin (30 μM). Upper panels show cells stained with fluorescent brightener 28 to reveal bud scars. Lower panels display the Ssa1‐GFP signal. Scale bar = 5 μm. (d) Percentage of cells with an Ssa1‐GFP focus. Mean ± SD. Dots represent independent experiments (N ≥ 163 cells). *p* values was calculated with an unpaired t test, **<0.01. (e) Percentage of cells exposed for 3 h to tripentadecanoin before or after aging with an Hsp104‐GFP focus. Mean ± SD. Dots represent independent experiments (N ≥ 124 cells). *p* values are adjusted *p* values from an ANOVA comparing to untreated old cells ****<0.0001. (f) Replicative lifespan analysis of yeast cells ±tripentadecanoin (30 μM). Age is expressed as the area of microcolonies normalized to the median of untreated cells. N ≥ 379 microcolonies. *p*‐value was obtained from a log‐rank (mantel‐cox) test. g. Heat map of the differentially expressed genes ± tripentadecanoin (30 μM). Genes presented have a |log2(tripentadecanoin.Treatment‐control)| ≥ 0.5 and a *p*‐value <0.05 (two‐tailed t test). Dubious open reading frames were removed from this list. The volcano plot of the whole set is presented as Figure S2 and the full heat map is presented as Figure S3

To understand the mode of action of tripentadecanoin, we switched to *S. cerevisiae* as a model organism. We used the age‐induced protein deposit as a readout for activity of tripentadecanoin counteracting the formation and toxicity of damaged and misfolded proteins. We obtained approximately 10 generations old cells expressing Hsp104‐GFP from its endogenous locus, cultured in liquid media. Remarkably, old cells exposed to 1 μM, 10 μM and 30 μM tripentadecanoin throughout the aging process were less prone to display a Hsp104‐GFP labelled protein aggregate, in a dose‐dependent manner, than untreated cells of the same age (Figure 2a–b). We observed a similar result with a whole extract from *Ophioglossum* (10 μg/ml). Tripentadecanoin also reduced the proportion of cells displaying an age‐induced protein aggregate detected in cells expressing Ssa1‐GFP (Figure 2c–d), arguing that tripentadecanoin does not target Hsp104‐GFP recruitment to these aggregate but readily prevent their formation. Interestingly, exposure of young cells to tripentadecanoin (30 μM) for 3 h at the beginning of the experiment led to a similar inhibition of protein aggregate formation as when cells are exposed throughout their aging (Figure 2e). In contrast, exposure of old cells to tripentadecanoin (30 μM) for 3 h just before imaging did not reduce the proportion of cells with Hsp104‐GFP foci (Figure 2e). Remarkably, prevention of the formation of age‐induced protein aggregate was accompanied by an increase in replicative lifespan (Figure 2f).

**FIGURE 3:**
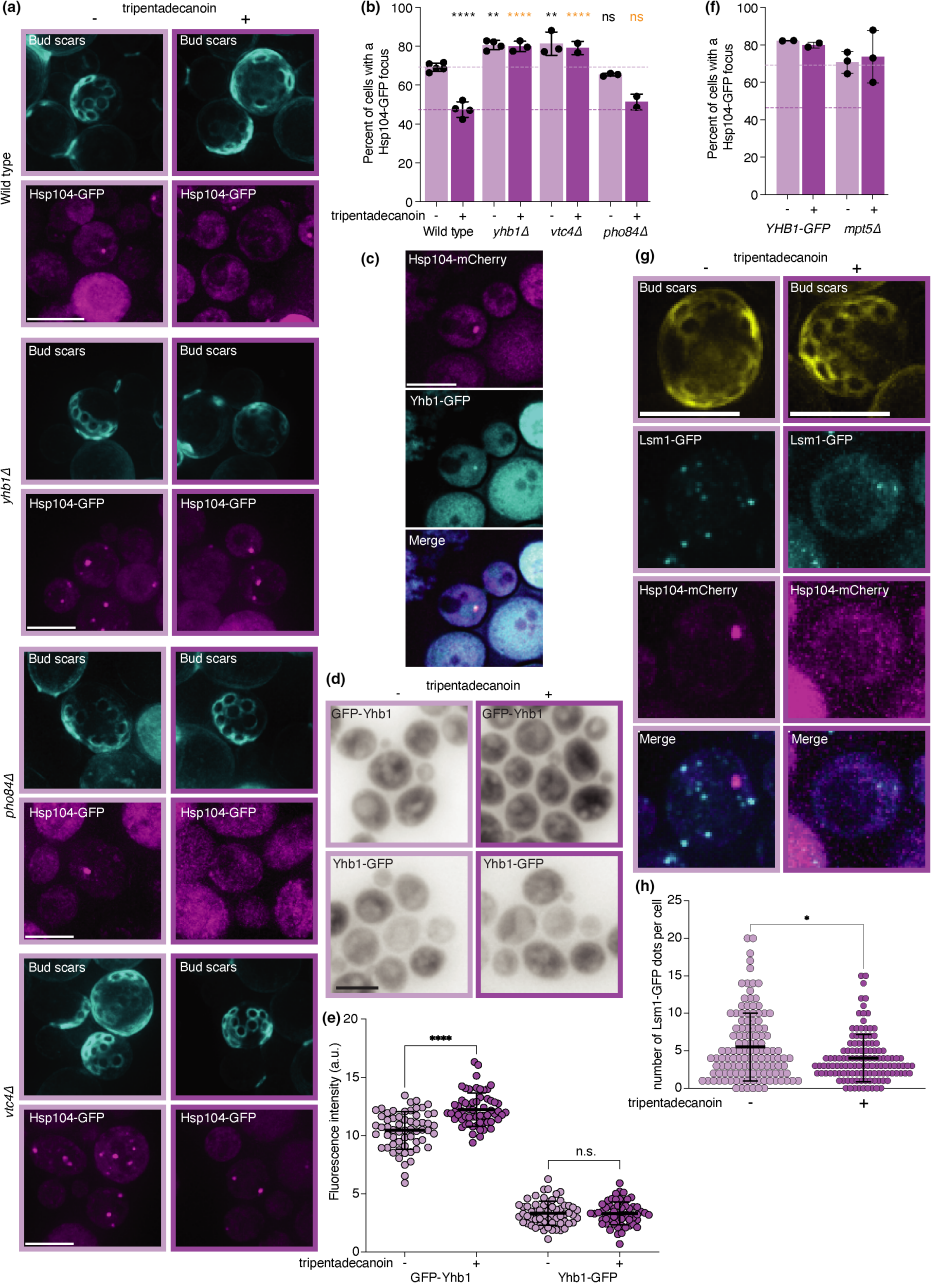
YHB1 mRNA regulation by P‐bodies is required for tripentadecanoin to prevent the formation of age‐induced protein deposits. (a) Representative images of old cells ±tripentadecanoin (30 μM) of indicated genotypes. Bud scars stained with fluorescent brightener 28 (upper panels). Hsp104‐GFP fluorescence signal (lower panels). Scale bars = 5 μm. (b) Percentage of cells with an Hsp104‐GFP focus in the indicated genotypes. Mean ± SD. Dots represent independent experiments (N ≥ 211 cells). *p* values are adjusted *p* values from an ANOVA comparing to wild type control (black stars) or to wild type treated with tripentadecanoin (orange stars). **<0.01; ****<0.0001; ns = not significantly different. (c) Colocalization of Yhb1‐GFP and Hsp104‐mCherry foci in old cells. Scale bar = 5 μm. (d) Representative sum projection images of cells expressing Yhb1‐GFP or GFP‐Yhb1 ± tripentadecanoin. Scale bar = 5 μm. (e) Quantification of GFP‐Yhb1 and Yhb1‐GFP mean fluorescence intensity ±30 μM tripentadecanoin for 5 h. Mean ± SD, N = 60 cells. *p*‐values obtained from unpaired t tests. ****<0.0001; ns = not significantly different. (f) Percentage of cells with an Hsp104‐GFP focus in the indicated genotypes. Mean ± SD. Dots represent independent experiments (N ≥ 188 cells). Dotted lines correspond to the WT ± tripentadecanoin from Figure 3b. (g) Representative images of old cells ±30 μM tripentadecanoin with bud scars stained with fluorescent brightener 28 (upper panels) and Lsm1‐GFP (middle top panels) and Hsp104‐mCherry (lower middle panels) signals and the merge (lower panels). Scale bar = 5 μm. (h) Quantification of the number of Lsm1‐GFP foci in old cells from Figure 3g. *p*‐value = 0.0277 (*) obtained from a Mann–Whitney test (N > 123 cells)

The formation of these naturally occurring protein deposits is thus prevented by tripentadecanoin and allowed us to probe the mode of action of this compound. To identify genes that are differentially expressed in the presence of tripentadecanoin and thereby possibly confer its cytoprotective and lifespan extending effects, we performed an RNAseq analysis (Figure 2g, Figures [Link], [Link], Tables [Link], [Link]). Out of 5968 detected mRNAs, 53 genes were differentially expressed between untreated and tripentadecanoin‐treated cells, of which 33 are functional genes (62.3%) and 20 dubious open reading frames (37.7%). Affected genes seemed to be more prone to be regulated by the SAGA complex (Spt‐Ada‐Gcn5 acetyltransferase) than TFIID (*p* < 0.00001, Chi‐square test). Gene ontology analysis revealed that polyphosphate metabolic process (*p* = 0.008), pyridine‐containing compound metabolic process (*p* = 0.014), cellular response to oxidative stress (*p* = 0.02) and response to oxidative stress (*p* = 0.05) were significantly enriched in the differentially expressed functional genes (Table S2). Notably, this included *YHB1,* the yeast orthologue of human neuroglobin, several genes related to phosphate metabolism (*PHO5*, *PHO84* and *PHO8*), inorganic polyphosphate synthesis and transport (*VTC3*). To further test the involvement of these genes in preventing the formation of age‐induced protein deposits, we used knock‐out strains of selected genes that included *YHB1*, *VTC4* and *PHO84. VTC4* was chosen because it encodes the vacuolar membrane polyphosphate polymerase instead of a regulatory subunit. Compared to wild type old cells, more *yhb1*Δ and *vtc4*Δ old cells contained an Hsp104‐GFP focus, while *pho84*Δ old cells were similar to wild type old cells (Figure 3a–b). In addition, *yhb1*Δ and *vtc4*Δ cells typically contained multiple Hsp104‐GFP foci (Figure 3a). These results suggest that both Yhb1 and Vtc4 counteract the formation of age‐induced protein deposits. We next tested whether tripentadecanoin was still preventing the formation of age‐induced protein deposit in the mutant strains. While tripentadecanoin (30 μM) reduced the percentage of old wild type and *pho84*Δ cells with a Hsp104‐GFP focus, this effect was lost in *yhb1*Δ and *vtc4*Δ cells (Figure 3a–b). Thus, we conclude that both Yhb1 and Vtc4 are essential for the protective effect of tripentadecanoin. We further focused on Yhb1 because its mammalian orthologue, neuroglobin, is identified, while Vtc4's orthologue is not.

We next tested whether Yhb1 is recruited to the age‐induced protein deposit by obtaining old cells that express Yhb1‐GFP and Hsp104‐mCherry from their endogenous loci. 69.1% of the Hsp104‐mCherry foci also recruited Yhb1‐GFP (Figure 3c). This recruitment was not affected by treatment with 30 μM tripentadecanoin (66.7% of the Hsp104‐mCherry foci recruited Yhb1‐GFP). We then asked if and how tripentadecanoin could induce *YHB1* expression. *YHB1* mRNA is destabilized by the Pumilio family proteins (Puf), particularly Mpt5 (Puf5) (Russo & Olivas, 2015). This regulation occurs through two overlapping Puf recognition elements in the 3'untranslated region of *YHB1* mRNA. To test if the 3'UTR of *YHB1* was required for its induction by tripentadecanoin, we used the C‐terminally tagged version of *YHB1* which replaces the native 3'UTR with the *ADH1* terminator (Yhb1‐GFP) and a strain expressing a N‐terminally tagged *YHB1* under the control of the strong *GPD* promoter (Janke et al., 2004) with the native *YHB1* terminator (GFP‐Yhb1). After 5 h of exposure to tripentadecanoin (30 μM) expression of GFP‐Yhb1 was induced but not of Yhb1‐GFP (Figure 3d‐e). Indeed, tripentadecanoin did not reduce the percentage of cells with an age‐induced protein deposit in the Yhb1‐GFP strain (Figure 3f). To test how tripentadecanoin induces *YHB1* expression further, we knocked out *MPT5* and obtained old cells expressing Hsp104‐GFP. While *mpt5*Δ old cells harboured as many age‐induced protein deposits as old wild type cells, tripentadecanoin had no effect on these cells (Figure 3f). These results suggested that induction of *YHB1* expression occurs at the level of its mRNA processing and prompted us to analyse the localization of p‐bodies in old cells with or without tripentadecanoin treatment. We tagged the p‐body component Lsm1 with GFP and observed bright foci as described (Hu et al., 2018). However, after tripentadecanoin treatment, less Lsm1‐GFP p‐bodies were assembled in old cells (Figure 3g–h), suggesting that tripentadecanoin affects p‐bodies regulation of *YHB1* mRNA decay/availability for translation in budding yeast.

**FIGURE 4:**
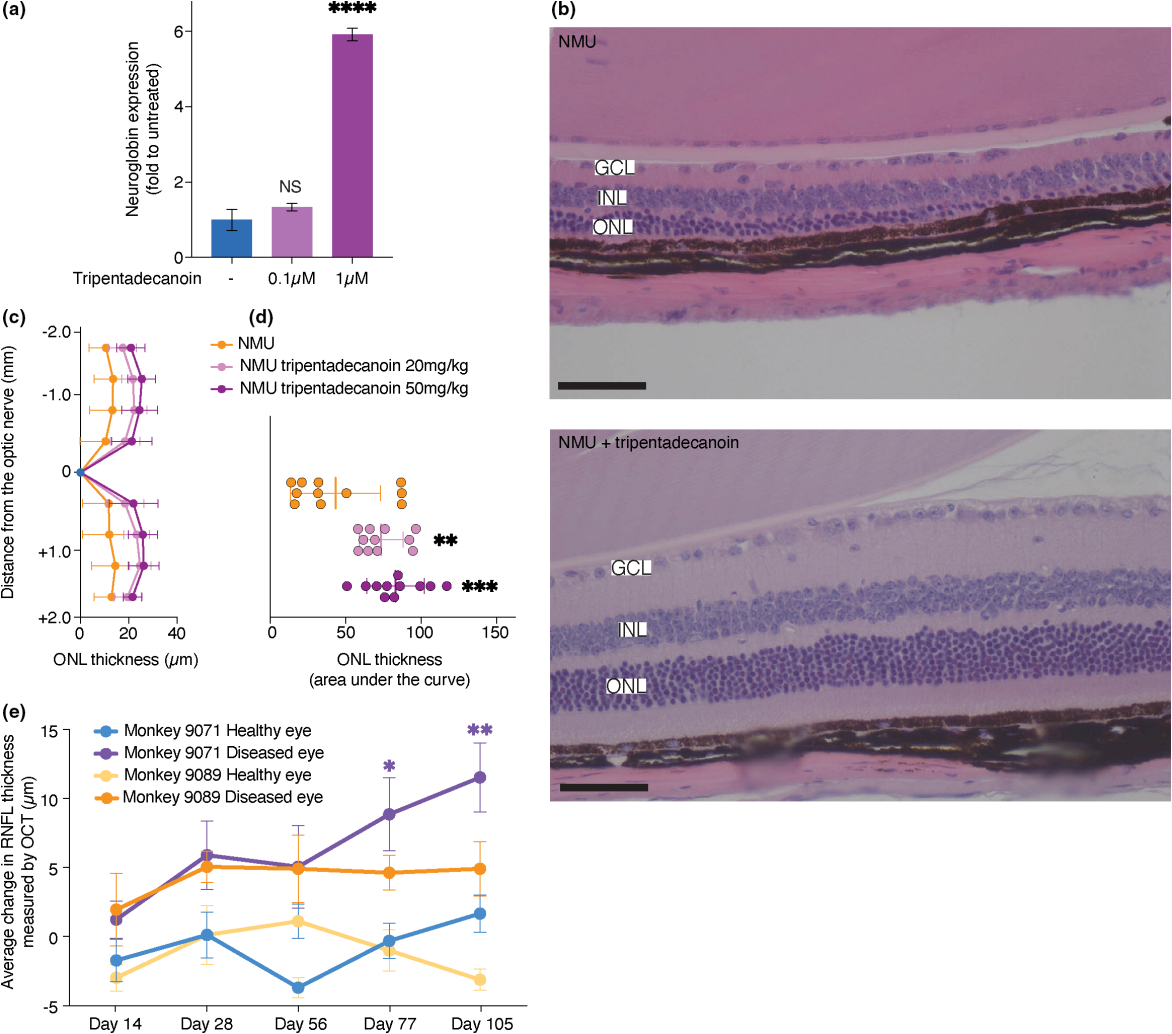
Tripentadecanoin induces neuroglobin expression in mouse primary cortex neurons and rescues N‐nitroso‐N‐methylurea (NMU)‐induced photoreceptor damage in mice. (a) Expression of neuroglobin mRNA over the control Rps28 mRNA presented as fold increase over untreated cells. *p* values are adjusted *p* values from an ANOVA comparing to untreated cells. ****<0.0001 (b) haematoxylin‐ and eosin‐stained retinal sections of eyes from mice treated with NMU (top) or NMU + Tripentadecanoin (bottom). ONL: Outer nuclear layer, INL: Inner nuclear layer, GCL: Ganglion cell layer. Scale bar = 50 μM (c). outer nuclear layer thickness measured from the retina sections as a function of the distance to the optical nerve (mm). Mean ± SD are presented. (d) Outer nuclear layer thickness presented as the area under the curve from panel c. *p* values are adjusted *p* values from an ANOVA comparing to NMU‐treated cells. ** < 0.005; *** < 0.001; **** < 0.0001. (e) Average change of retinal nerve fibre layer measured by OCT in monkeys #9071 and #9089 treated with tripentadecanoin. In each case, the left healthy eye and right diseased eye were measured. Averages within an eye were compared to the data before treatment with an ANOVA. *<0.05, **<0.005

To test whether tripentadecanoin also induces neuroglobin in mammalian cells, we measured the mRNA levels of neuroglobin in mouse primary cortex neurons. After 3 h of treatment, neuroglobin mRNA level reached to 1.34 folds of the control for a 100 nM treatment and 5.91 folds for a 1 μM treatment (Figure 4a). These results strongly suggest that mRNA levels of neuroglobin are regulated similarly to *YHB1* in response to tripentadecanoin. To test the effect of tripentadecanoin in vivo, we chose an N‐nitroso‐N‐methylurea (NMU) induced photoreceptor degeneration model for several reasons: Yhb1 has mostly been associated with nitrosated stress (Lewinska & Bartosz, 2006) while over‐expression of neuroglobin was previously shown to rescue visual defects induced by NMU (Tao et al., 2017). Moreover, NMU causes photoreceptor degeneration within 7 days of a single‐dose injection and the induced damage mimics the photoreceptor degenerative process in progressive human retinal degenerative diseases. Mice treated with NMU and tripentadecanoin (20 mg/kg or 50 mg/kg) displayed a significant protection of the outer nuclear layer of the retina compared to mice treated only with NMU (Figure 4b–d) in agreement with the prior mode of action studies (Tao et al., 2017). Furthermore, Harlequin mutant mice that display retinal ganglion cell loss and optic atrophy, due to a respiratory chain complex I defect, have a 2‐fold reduced neuroglobin expression. Neuroglobin overexpression in harlequin mice eyes rescued retinal ganglion cell (RGC) body number and RGC axon number (Lechauve et al., 2014). Interestingly, following tripentadecanoin treatment in two rhesus monkeys (*Macata mulatta*) with unilateral optic atrophy (from a family with optical atrophy and retinal vascular abnormalities history, see material and methods), an increase in the average thickness of Retinal Nerve Fibre Layer (RNFL) was observed in the eyes with optic atrophy, but not in the healthy eyes (Figure 4e).

## 3 DISCUSSION

Our results provide evidence that tripentadecanoin induces the expression of *YHB1* in budding yeast to prevent the formation of age‐induced protein aggregates. Yhb1 is known to be involved in the response to nitrosative stress (Liu et al., 2000). Yhb1 catalyses the reaction of NO• with oxygen to create nitrate, limiting exposure of the cell to NO• (Liu et al., 2000). When exposed to nitrosating agents, *yhb1*Δ cells accumulate nitrosylated proteins (Liu et al., 2000). Therefore, our results suggest that the damaged proteins accumulating in the age‐induced protein deposits could include nitrosylated proteins and that damages induced by NO• may be limiting yeast lifespan.

Yhb1 also protects against the toxicity of heterologous α‐Synuclein overexpression in yeast and the mitochondrial fragmentation associated with it. Interestingly, exogenously expressed neuroglobin can rescue the deletion of yeast *YHB1* demonstrating a conservation of function between the two orthologues (Kleinknecht et al., 2016). Supporting this conservation, we found that tripentadecanoin induces the expression of neuroglobin, protects or rescues cells against toxic amyloids and prevents NMU‐induced photoreceptor damage in mice and optic atrophy in Rhesus monkeys. Neuroglobin induction was previously shown to be protective during hypoxic–ischaemic insults (Sun et al., 2001), to have cytoprotective effects against α‐synuclein (Kleinknecht et al., 2016) and amyloid‐β toxicity (Khan et al., 2007) and to inhibit apoptosis (Guidolin et al., 2016). Accordingly, its levels are increased in Alzheimer patients at early/moderate stages of the disease but decreased in severe cases (Sun et al., 2013). Neuroglobin appears as a central target to prevent different forms of neuronal degeneration. With tripentadecanoin, we have identified a small molecule that may protect patients against neurodegeneration of the retina as well as in proteinopathies.

Finally, an attractive hypothesis for the role of the VTC complex and phosphate metabolism is their link to the production and transport of polyphosphates in and out of the vacuole (Hothorn et al., 2009). The vacuole is the equivalent of lysosomes in human cells and recent work suggests that the lysosome may be playing an important role in managing protein deposits in neural stem cells (Leeman et al., 2018). Therefore, lysosomes and the vacuole in yeast emerge as potential regulators of age‐induced protein aggregation and aging. Vacuolar functions are known to be less efficient in old cells, because its internal pH raises (Henderson et al., 2014; Hughes & Gottschling, 2012) and impact mitochondrial activity (Hughes et al., 2016; Hughes & Gottschling, 2012; Veatch et al., 2009). Inorganic polymers of phosphates have been found to work molecularly as primordial chaperones (Gray et al., 2014) that could counteract the toxic effects of age‐induced protein aggregates (Cremers et al., 2016). Thus, regulating the levels of inorganic polyphosphates in the aging cell may contribute to how well it will age. It will be important in the future to understand if inorganic polyphosphates quantity is changed upon tripentadecanoin treatment in diverse model organism, during aging and this may as well help us to identify the elusive mammalian inorganic polyphosphate polymerase.

## 4 EXPERIMENTAL PROCEDURES

### 4.1 Preparation of *Ophioglossum* whole extract

1 ml of DMSO was added to 10 mg of *Ophioglossum* in a 1.5 ml eppendorf tube. The eppendorf tube was rotated overnight at a temperature of 30–37°C. Appropriate amounts of the supernatant were added to culture media.

### 4.2 Preparation of tripentadecanoin

Tripentadecanoin (C_48_H_92_O_6_) was obtained from Sigma‐Aldrich (T4257). 5 mg of the powder was resuspended in 0.6 ml of pre‐warmed (35°C) 100% ethanol and vortexed for 5 min at room temperature. The stock solution was sonicated in a water bath at 35°C for 30 min and appropriate amounts were added to culture media.

### 4.3 Yeast strains

The strain used for RNAseq is s288c BY4741 wild type (yFC01: *MAT* **a**, *his3*Δ*1, leu2*Δ*0, ura3*Δ*0, met15*Δ*0, ADE2, TRP1*). Strains to obtain old cells were derived from the Mother Enrichment Program (Lindstrom & Gottschling, 2009) strain expressing Hsp104‐GFP from its endogenous locus with deletions and *GPD*_*prom*_ *‐GFP‐YHB1* strains were obtained according to (Janke et al., 2004) and are listed in Table S3.

### 4.4 Obtention of old yeast mother cells

Exponentially growing cells were diluted to OD_600nm_ of 0.02 in 25 ml SC‐Full containing 1 μM beta‐oestradiol (Sigma‐Aldrich E8875) and tripentadecanoin or *Ophioglossum* whole extract, or 75 μl ethanol as a control. The yeast cultures were incubated in a shaking incubator at 30°C for 18–22 h. Cells were pelleted (600 g, 2 min) and resuspended in 1 ml SC‐Full and supplemented with 10 μl Fluorescent Brightener 28 (Sigma‐Aldrich F3543). Cells were incubated for 5 minutes in the dark and pelleted (600 g, 1 minute), washed twice in 1 ml SC‐Full and finally resuspended in 500 μl SC‐Full. 10 μl of the cells were placed on a SC‐Full agar pad and imaged with a DeltaVision Elite microscope equipped with a sCMOS camera. Images were deconvolved using SoftWorx (GE Healthcare). For Ssa1‐GFP old cells, images were acquired using a Nikon inverted microscope equipped with an Andor Dragonfly spinning disk and an EMCCD iXon888 (Life Andor). Image analysis was performed using FIJI (Schindelin et al., 2012).

### 4.5 Quantification of Yhb1‐GFP and GFP‐Yhb1

Exponentially growing cells were diluted in SC‐Full ±tripentadecanoin (30 μM) and grown at 30°C for 5 h before imaging with a DeltaVision Elite microscope equipped with a sCMOS camera. Z‐stacks were sum‐projected and background was subtracted. Mean fluorescence intensity in the whole was measured. Note that GFP‐Yhb1 is under a strong promoter, explaining the fluorescence level difference with Yhb1‐GFP.

### 4.6 Quantification of Lsm1‐GFP foci in old cells

Z‐stacks were max‐projected and background was subtracted. All images were similarly thresholded and foci were counted in each old cell.

### 4.7 RNAseq

Exponentially growing cells were diluted to OD_600nm_ 0.2 and grown for 5 h ±tripentadecanoin (30 μM) in triplicate. Cells were pelleted and plunged in liquid nitrogen. RNA extraction, library preparation and Illumina HiSeq were performed by Genewiz®. F (Watson) and R (Crick) reads were aligned to the S. cerevisiae using BLAT and the read count distribution was determined for each dataset. Read counts per bp from each dataset were normalized by dividing them by the corresponding average genomic read count. Normalized F and R read counts for each gene were then averaged and aligned by their transcription start site (Xu et al., 2009). The median read count for each gene (from the transcription start site to the end of the coding sequence) was then determined for each transcript. Intron regions were excluded from the calculation. We found 415 genes whose expression was significantly affected by the drug using a two‐tailed t test (*α* = 0.05) between the control and drug‐treated cells (three biological replicates each) and 53 genes out of that set had an average |log2(drug. treatment‐control)| ≥ 0.5. 20 of these genes are annotated as dubious or uncharacterized on the Saccharomyces Genome Database (SGD, www.yeastproteome.org). Gene Ontology analysis was performed on the SGD website with a *p*‐value of 0.05 (Table S2). Raw counts are presented in Table S1, with TFIID and SAGA control of gene expression determined using reference from (Huisinga & Pugh, 2004).

### 4.8 Yeast replicative aging

Lifespan analysis was assayed as described by Moreno et al. (Moreno et al., 2019). Briefly, wild‐type MEP strain was cultured overnight in SC‐Full and diluted to OD600 of 0.2 in 25 ml SC‐Full containing 1 μM β‐oestradiol. Tripentadecanoin (30 μM) was then added or omitted and incubated for 5 h at 30°C, 200 rpm. The culture was then diluted to OD600 of 0.01 and 500 μl of this dilution was plated on YPD containing 1 μM β‐oestradiol. Plates were incubated at 30°C for 4 days. Microcolonies were imaged using a Nikon Eclipse 50i microscope with a 10X/0.25 Nikon plan dry objective. Areas of the microcolonies were determined as in Moreno et al. (Moreno et al., 2019) using FIJI. Data were normalized to the median of the untreated condition.

### 4.9 Amyloid oligomers

Aβ_1‐42_ was obtained from Bachem (ref H1368). PrP_118‐135_ was obtained from Bachem (ref H‐4206). Human wild‐type recombinant α‐synuclein was obtained from r‐Peptide (ref 0101008603). Human wild‐type recombinant tau (2N4R) protein was obtained from Evotec. Amylin was obtained from Bachem (ref H‐7905.1000).

### 4.10 Mouse primary cortex neurons

These experiments were performed by SynAging SAS on behalf of SunRegen Heathcare AG.

#### 4.10.1 Cell culture

Cortical neurons from embryonic day 16–17 were prepared from C57Bl6/J mouse foetuses, as previously described (Pillot et al., 1999). Dissociated cortical cells were plated (50.000 cells/well) in 48‐well plates pre‐coated with 1.5 μg/mL polyornithine (Sigma). Cells were cultured in a chemically defined Dulbecco's modified Eagle's/F12 medium free of serum and supplemented with hormones, proteins and salts. Cultures were kept at 35°C in a humidified 6% CO2 atmosphere.

#### 4.10.2 Challenging cells with amyloid oligomers and MTT assay on cortical neurons pre‐incubated with *Ophioglossum* extract or tripentadecanoin

Before addition of vehicle or an amyloid oligomer, neurons were pre‐incubated at DIV 4 with various concentrations of *Ophioglossum* extract or Tripentadecanoin for 48 h in fatty acid free medium. At DIV 6, medium was removed and cells were incubated for 24 h with vehicle or 1,0 μM AβO in a final medium volume of 120 μl per well. For positive control, cells were pre‐incubated with 0.05 μM DHA‐ethyl ester (Sigma, D2410) for 48 h before vehicle or an amyloid oligomer treatment. Cells were incubated for 24 h before monitoring cell viability using the MTT assay: cells were incubated at 35°C for 1 h with MTT (Sigma, Cat #M2128‐10G). For that purpose, 14 μl of 5 mg/mL MTT (solubilized in PBS) were added to each well. After incubation, medium was removed, and cells were lysed with 150 μl DMSO for 10 minutes and protected from light. After complete solubilization of formazan, absorbance at 570 nm was recorded using a Spectrophotometer BMG Labtech Fluostar Omega. All treatments were done in triplicate.

#### 4.10.3 MTT assay on cortical neurons incubated with *Ophioglossum* extract or Tripentadecanoin at the same time or after cells were challenged with amyloid oligomers

Neurons were incubated in fatty acid free medium with vehicle or an amyloid oligomer in the absence or presence of increasing concentrations of Tripentadecanoin added concomitantly to the amyloid oligomer, 3 h or 6 h after. Cells were incubated for 24 h in a final volume of 140 μl per well. For positive control, cells were treated similarly in the presence of 0.1 μM HNG (S14G variant of humanin peptide). Cell viability was monitored using the MTT assay. Cells were incubated at 35°C for 1 h with MTT (Sigma, Cat #M2128‐10G, Lot # MKBH7489V). For that purpose, 14 μl of 5 mg/mL MTT (solubilized in PBS) were added to each well. After incubation, medium was removed and cells were lysed with 150 μl DMSO for 10 minutes and protected from light. After complete solubilization of formazan, absorbance at 570 nm was recorded using a Spectrophotometer BMG Labtech Fluostar Omega. All treatments were done in triplicates.

#### 4.10.4 RT‐qPCR

Total RNA samples were extracted from lysates of mouse primary cortex neurons. cDNA were obtained with the Transcriptor Reverse Transcriptase kit from Roche. PCR were performed using the LightCycler system (Roche Molecular System Inc.) according to the supplier's instructions. Transcripts analysis was done in triplicate using the primers CCCTATCTATGTGTGTCTG (forward) and TGAGGACCAAGGTATAGA (reverse) and the probe ATCTGCCTGTTGTAGTCTTAGCCTC for Neuroglobin. Data were normalized using the Rps28 gene as a control.

### 4.11 Human‐induced pluripotent stem cells

These experiments were performed by SynAging SAS on behalf of SunRegen Heathcare AG.

Cells (HIP‐Neuronal progenitors, GlobalStem, Cat#GSC‐4312) were plated in 96‐well plates at a density of 60.000 cells per well and culture according to supplier's recommendations. Before experiments, cells were matured for 5 weeks and kept at 37°C in a humidified 5% CO2 atmosphere. Cells were incubated with vehicle or 1 μM AβO in the absence or presence of increasing concentrations of tripentadecanoin added concomitantly to AβO (T0), 3 h after AβO (T3), or 6 h after AβO (T6). Cells were incubated for 24 h in a final volume of 100 μl per well. For positive control, cells were treated similarly in the presence of 0.1 μM HNG (S14G variant of humanin peptide). Neuronal loss was monitored using the detection of neuronal‐specific enolase by ELISA assay according to the supplier's recommendations (CloneCloud, Cat#SEA537Hu). A total of three data points per experimental condition were generated.

### 4.12 N‐Nitroso‐N‐methylurea

These experiments were performed by IRIS PHARMA and Prof. Heping Xu at the School of Medicine, Dentistry & Biomedical Science, Queen's University Belfast, 97 Lisburn Road, Whitla Medical Building BT9 7BL Belfast, United Kingdom on behalf of SunRegen Healthcare AG. The experimental phase performed at the animal facility of Queen's University Belfast (UK) was conducted in accordance with the ARVO Statement for the Use of Animals in Ophthalmic and Vision Research, and the study was approved by the local Animal Welfare Ethical Review Body (AWERB). NMU was obtained from Fluorochem (90%: 10% stabiliser [Acetic acid]. Batch number: FCB013586). Animals were housed with one to five mice in each cage. All animals were maintained under a 12‐h light and dark‐controlled cycle. Temperature and relative humidity were maintained at 22 ± 2°C and 60 ± 10% respectively. Throughout the study, animals had free access to food and water. The mice were anaesthetized via intra peritoneal injection of ketamine hydrochloride (60 mg/kg, Vetoquinol UK Ltd, Northamptonshire) and xylazine hydrochloride (5 mg/kg, Bayer HealthCare, KVP pharma). The NMU solution in acetic acid (source of acetic acid) was diluted in nuclease free water (source of the nuclease‐free water) to obtain a solution at 6.25 mg/mL (0.008% acetic acid) just before use. Mice received one intraperitoneal injection of NMU at a dose of 50 mg/kg (8 ml/Kg). Eighteen 8‐ to 12‐week‐old female C57BL/6J mice were randomized into three groups: (1) NMU + vehicle; (2) NMU + tripentadecanoin 20 mg/kg and (3) NMU + tripentadecanoin 50 mg/kg. NMU at a dose of 50 mg/kg was injected intra peritoneally to all mice; tripentadecanoin (or vehicle) was administered daily via oral gavage starting 3 days before NMU and continuing until 7 days after NMU challenge. Animals were euthanized by inhalation of CO2. After euthanasia, both eye from each animal were collected, fixed in Davidson's solution (0.08% paraformaldehyde PFA) over‐night at room temperature, rinsed in 70% ethanol for 3 h at room temperature, and stored at 5° ± 3°C. Both eyes from each animal were embedded in paraffin for histological analysis. Paraffin sections (5–7 μm thick) were performed along the vertical meridian and stained with haematoxylin/eosin stain. The vertical meridian included the optic nerve. Three sections per eye were examined under a standard microscope (Leica). outer nuclear layer thickness was measured every 500 μm (four points) from the optic nerve to the peripheral retina in each region of the retina (superior and inferior) using a standard microscope (Leica) operated by a single observer masked to treatment. The thickness of the outer nuclear layer was measured at each point, and the number of rows of photoreceptor nuclei was quantified. Results were expressed as the outer nuclear layer (ONL) area under the curve (AUC_−1.75 to +1.75 μm_) and number of rows of photoreceptor nuclei.

### 4.13 Rhesus monkeys

Two male monkeys (*Macaca mulatta*) (monkey 9071, 5 years old, 6.6 kg; monkey 9089, 4 years old, 4.5 kg) had a family history of optic/retinal diseases and were selected into this study. Both animals did not show increased cup/disc ratio, nor increased intraocular pressure, nor increased blood glucose. They did not show glaucoma type local retinal nerve fibre layer thinness, but a general thinness in all regions of retinal nerve fibre layer. Both animals had unilateral optic atrophy localized to the right eye. Rhesus monkeys were treated with orally administered SBC003 at a dose of 5 mg/kg/day for 2 weeks, 15 mg/kg/day for 4 weeks, 30 mg/kg/day for 3 weeks, 2 weeks wash out period, 50 mg/kg/day for 4 weeks. The following parameters were evaluated during tripentadecanoin treatment: body weight, food consumption, clinical observations, clinical biochemistry and haematology, as well as ocular anterior and posterior examinations including assessment of interocular pressure, fundoscopy and ocular coherence tomography (OCT).

OCT in optic disc and macular regions: frequency: Month‐4, D‐8 and on D14, D28, D56, D77 and D105. Animals were anaesthetized with 1:1 Ketamine:Xylazine mix (6 mg/kg ketamine, intramuscular injection), two drops of Tropicamide Phenylephrine Eye Drop were applied to each eye after anaesthesia for pupil dilation. OCT image acquisition protocol: subject's forehead was kept leaned against the forehead support. Following image acquisition, RNFL thickness was measured after determining the distance between the anterior and posterior surface of RNFL. The anterior and posterior surface of RNFL was detected automatically by the built‐in software of Heidelberg OCT. Locating the posterior surface of RNFL manually was necessary and performed by two masked and experienced examiners. Once the anterior and posterior surface of RNFL was determined, built‐in software of Heidelberg OCT showed the RNFL thickness along the circumference around the optic disc (RNFL thickness in optic disc‐centred images) or the RNFL thickness in macular regions. Retinal nerve fibre layer was measured at seven different locations: global, nasal, nasal superior, temporal superior, temporal, temporal inferior and nasal inferior. The comparative data in the unaffected eyes served as an internal control to verify the reproducibility of OCT measurement in this study.

#### 4.13.1 General observations

During the study, the two monkeys were dosed orally. No.9071 cooperated well during the entire study; No.9089 refused to take apples from D99 and capsules were orally administered from D101. There was no major change in food intake or body weight in both monkeys. During the study, no drug‐related abnormalities were observed in animals. The food intake was within the variation of food intake at this age. At the beginning of capsule dosing at week 15, No.9089 food intake of week 15 decreased to 117 g possibly due to oral dosing change from apple food‐admix to capsule form, and then he fully recovered at week 16. No change was observed in biochemistry of these monkeys. Levels of ALT, AST and BUN of monkey 9089 were slightly higher than normal limits before administration. Levels of LDL‐c, ALT and ALP of monkey 9071 were slightly higher than normal limits before administration. There was no significant change in biochemistry parameters during the study. No change was observed of the haematology of monkeys. There was no significant change in CBC parameters during the study and CBC indicators were within reference range. A pharmacokinetic (PK) study was performed on pre‐dosing (8 days prior to tripentadecanoin dosing) and on Days 78, 105 at 2 h after tripentadecanoin dosing. For monkey 9071, single point PK results showed that the exposure of tripentadecanoin increased with the dose level on D78 and D105. Tripentadecanoin plasma concentrations were tested at 6.3 ng/ml at baseline; 42.1 ng/ml at 2 h after dosing on Day 78. After Day 78, the plasma concentration fluctuated between 47.6–86.4 ng/ml, showing a trend of increased exposure with an increased dose. For monkey 9089, the plasma concentration of tripentadecanoin was not increased with the increased dose. It was reduced to 27.1 ng/ml or close to baseline regardless of the increased dose.

All procedures in this protocol followed the Animal Welfare Act, the Guide for the Care and Use of Laboratory Animals, and the Office of Laboratory Animal Welfare, and approved by IACUC of Sichuan Primed Shines Bio‐tech Co., Ltd.

## AUTHOR CONTRIBUTIONS

H.P.O. data acquisition and analysis, manuscript editing; N.R.R. data acquisition and analysis; P.K. supervision, data analysis, manuscript editing; J.S. supervision, data analysis, manuscript editing; M.R‐L data analysis, manuscript editing; Y.D. supervision, data analysis, manuscript writing; F.C. supervision, data acquisition and analysis, manuscript writing.

## CONFLICT OF INTEREST

SunRegen Healthcare AG has deposited the patent WO2017211274A1.

F.C. declares to have received an honorarium from SunRegen Healthcare AG for consulting and research.

## Supporting information


Figure S1

Figure S2

Figure S3

Table S1

Table S2

Table S3

Appendix S1

## Data Availability

Data are available on request from the authors.
