# Supplementary figures and images for: A rare natural lipid induces neuroglobin expression to prevent amyloid oligomers toxicity and retinal neurodegeneration
Source: Aging Cell. 2022 Jun 3;21(7):e13645. doi: 10.1111/acel.13645 (PMC9282837; doi:10.1111/acel.13645)

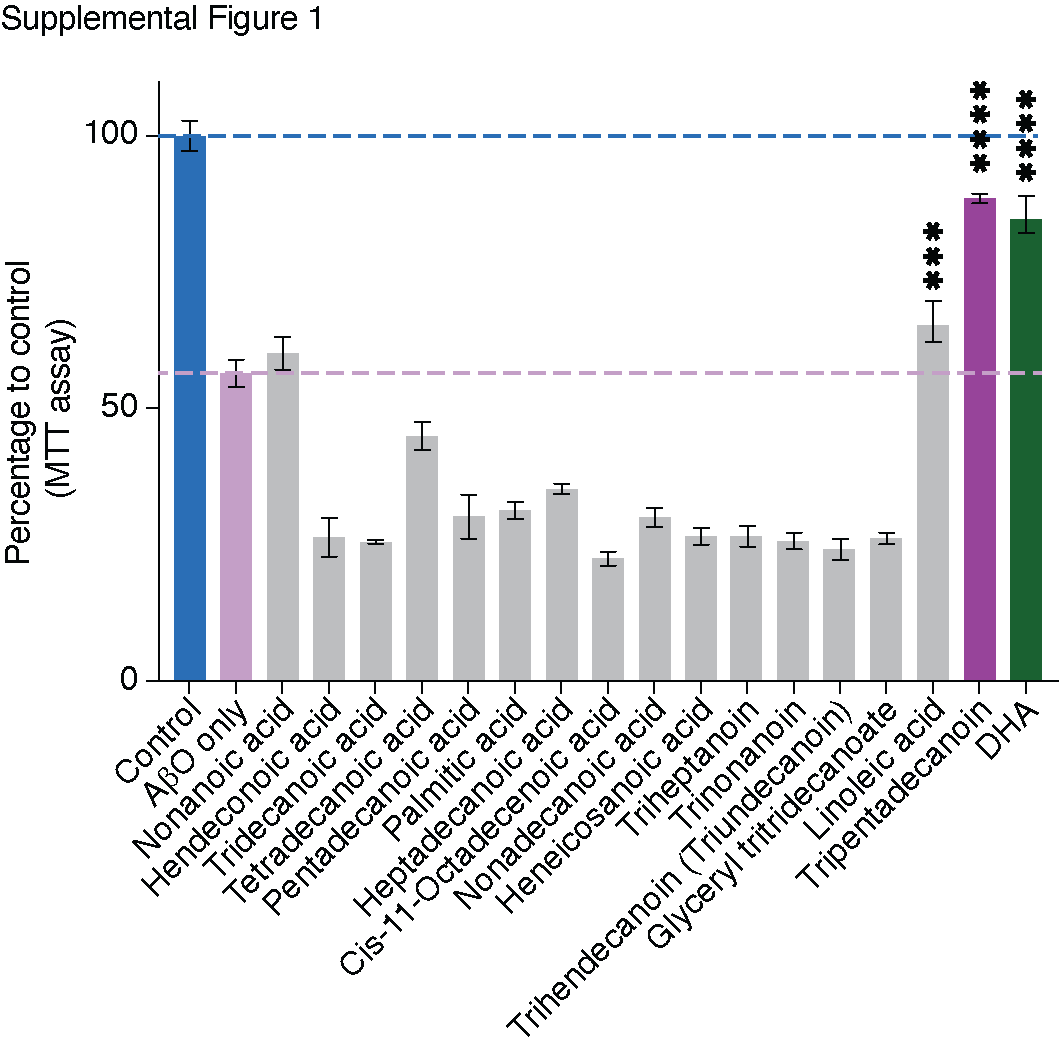

Supplement: Supplementary file 1 — Figure S1 [file ACEL-21-e13645-s007.tif]

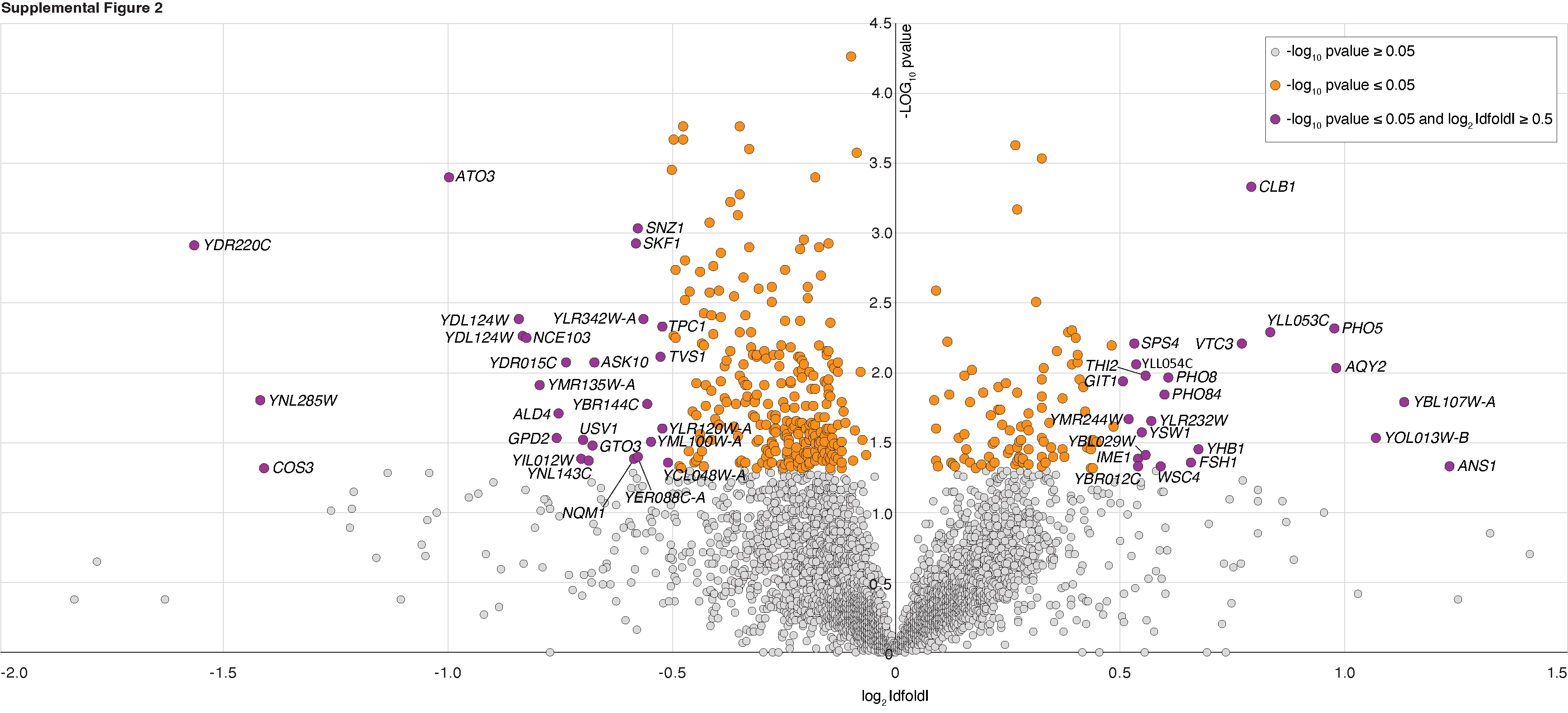

Supplement: Supplementary file 2 — Figure S2 [file ACEL-21-e13645-s004.tif]

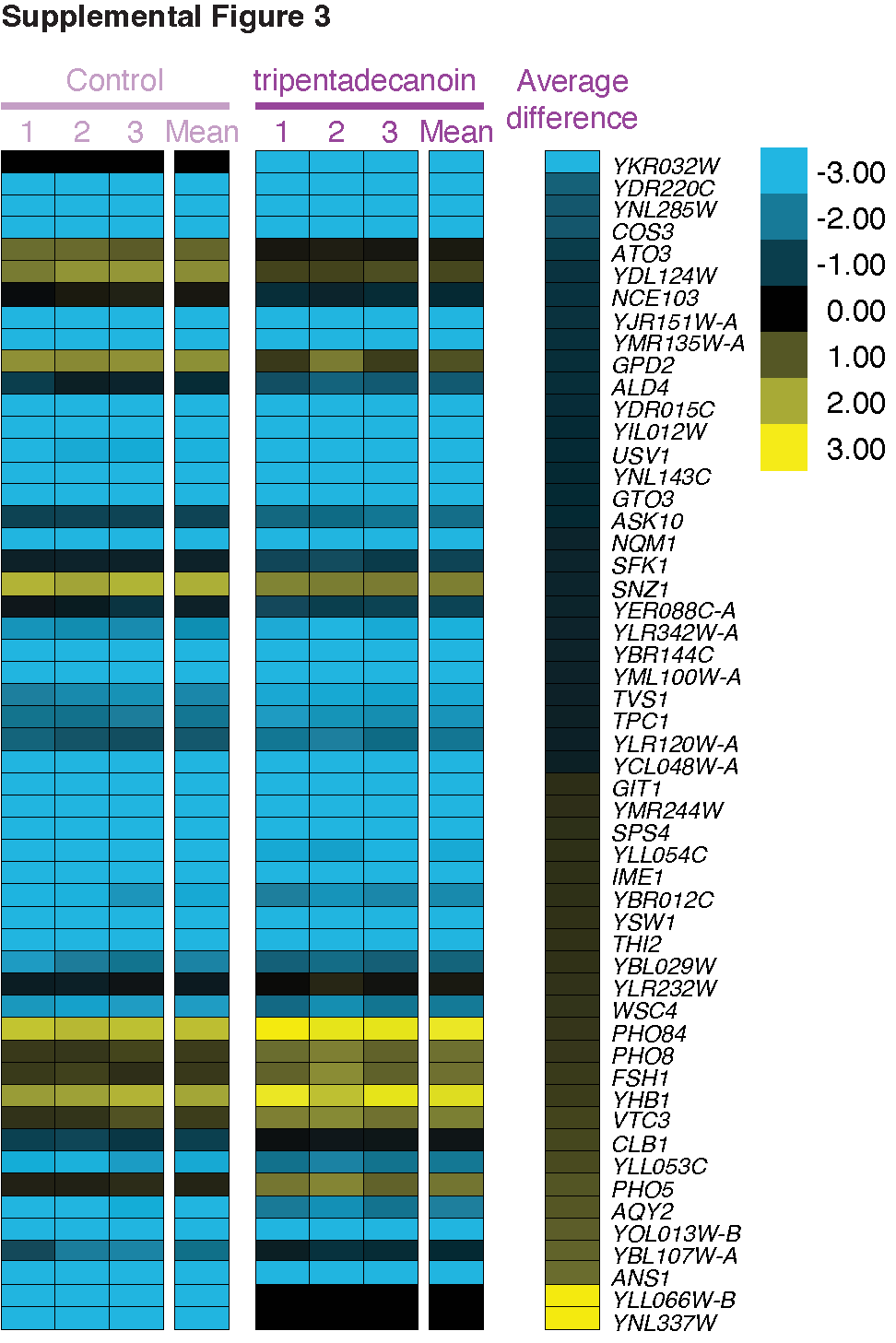

Supplement: Supplementary file 3 — Figure S3 [file ACEL-21-e13645-s002.tif]
