# Supplementary material for: A rare natural lipid induces neuroglobin expression to prevent amyloid oligomers toxicity and retinal neurodegeneration
Source: Aging Cell. 2022 Jun 3;21(7):e13645. doi: 10.1111/acel.13645 (PMC9282837; doi:10.1111/acel.13645)
Supplement: Supplementary file 7 — Appendix S1 [file ACEL-21-e13645-s001.docx]

**Supplemental Figure 1:** Quantification of mouse primary cortex neurons viability (MTT assay) after exposure to AβO (1µM) and pre-treatment with tripentadecanoin (1µM), DHA (50µM) or the indicated compounds at 1µM. P-values were obtained from ANOVA comparing to the toxin only treatment; ***<0.001; ****<0.0001.

**Supplemental Figure 2:** Volcano plot of the RNAseq data of differentially expressed genes. Gray dots are not significantly different in the two conditions, orange dots and significantly different and purple dots are both significantly different and have a log_2_ of the absolute difference fold ≥0.5.

**Supplemental Figure 3:** Heat map of the RNAseq hits including the dubious open reading frames.

**Supplemental Table 1:** RNAseq individual mRNA counts. TFIID and SAGA control of gene expression was determined using reference from (Huisinga & Pugh 2004).

**Supplemental Table 2:** List of Gene Ontology enrichments from the yeast RNAseq hits.

**Supplemental Table 3:** List of yeast strains.
